# Potential Applications of ^68^Ga-PSMA-11 PET/CT in the Evaluation of Salivary Gland Uptake Function: Preliminary Observations and Comparison with ^99m^TcO_4_^−^ Salivary Gland Scintigraphy

**DOI:** 10.1155/2020/1097516

**Published:** 2020-01-11

**Authors:** Yanhong Zhao, Yuxiao Xia, Huipan Liu, Zi Wang, Yue Chen, Wei Zhang

**Affiliations:** ^1^Department of Nuclear Medicine, Affiliated Hospital of Southwest Medical University, Luzhou, Sichuan 646000, China; ^2^Nuclear Medicine and Molecular Imaging Key Laboratory of Sichuan Province, No. 25, Taiping St., Luzhou, Sichuan 646000, China

## Abstract

**Purpose:**

To preliminarily evaluate the feasibility and potential of using ^68^Ga-PSMA-11 PET/CT in evaluating the function of salivary glands and lacrimal glands in comparison with ^99m^Tc-pertechnetate (^99m^TcO_4_^−^) salivary gland scintigraphy (SGS).

**Methods:**

A retrospective study was performed in 15 patients with different degrees of xerostomia and suspected salivary gland dysfunction. Each patient underwent ^68^Ga-PSMA-11 PET/CT first and SGS the next day, and the findings of both scans were compared.

**Results:**

The results of ^68^Ga-PSMA-11 PET/CT and SGS were consistent in 12/15 patients (80%) and were inconsistent in the remaining patients (20%). For 5 (33.3%) of 15 patients, ^68^Ga-PSMA-11 PET/CT provided more information than did SGS. Additionally, ^68^Ga-PSMA-11 PET/CT corrected the misdiagnosis by SGS for 1 patient.

**Conclusions:**

^68^Ga-PSMA-11 PET/CT is a potentially useful imaging tool for evaluating the function of salivary glands and lacrimal glands. ^68^Ga-PSMA-11 PET/CT can be a promising supplement to SGS, and its clinical value deserves further study.

## 1. Introduction

The salivary glands consist of three pairs of major salivary glands (parotid glands, submandibular glands, and sublingual glands) and multiple minor salivary glands that are widely distributed in the oral mucosa. Glandular function impairment occurs in approximately 60% of patients with xerostomia [1]. Dry mouth is frequently observed in salivary gland dysplasia, in obstructive diseases, in autoimmune syndrome, and in space-occupying lesions, with the help of radiotherapy and chemotherapy for head and neck tumors and with large-dose ^131^I treatment, and is most commonly observed in Sjogren's syndrome. According to the American-European Consensus Group (AECG) criteria, the diagnosis of salivary and lacrimal gland dysfunction is mainly based on the objective and subjective evaluations of patients [2]. However, most of the current studies use objective indicators as a more convincing means of evaluation because the subjective symptoms of patients do not necessarily reflect the dysfunction of salivary glands [3, 4].

The objective evaluation methods of clinical salivary gland function mainly include saliva analysis, radiography of the parotids, and ^99m^Tc-pertechnetate (^99m^TcO_4_^−^) salivary gland scintigraphy (SGS). The first two methods are affected by physiological factors, and their application is complicated; therefore, they are not ideal for clinical use. SGS allows a noninvasive inspection of the condition and has the advantages of simplicity, safety, and relatively low cost [5]. SGS is a commonly used method for the clinical evaluation of salivary gland function and has high specificity and sensitivity [6]. SGS has been generally proposed to assess salivary gland function in different indications [7, 8]. SGS can be used to directly observe the morphology and function of the bilateral parotid and submandibular glands. Image analysis for SGS depends on the observer and lacks uniform standards [9, 10].

Over the past few years, prostate-specific membrane antigen- (PSMA-) targeted radiotracers have been widely used in the diagnosis (^68^Ga-PSMA) and treatment (^177^Lu-PSMA) of prostate cancer. Among them, PSMA-targeted positron emission tomography (PET), especially ^68^Ga-PSMA-11, has been widely used for the detection, staging, posttreatment efficacy evaluation, and recurrence assessment of prostate cancer [11–13]. However, the binding of PSMA ligands is not limited to prostate cancer cells, and their physiological distribution includes the lacrimal glands, salivary glands, kidneys, duodenum, and small intestine [14]. Salivary glands and lacrimal glands have high uptake levels [14–17]. ^68^Ga-PSMA PET/CT can enable the visualization of head and neck lacrimal glands, primary and secondary salivary glands, and seromucous glands. All normal glands have increased tracer uptake [16]. A recent study by Rupp found that the normal submandibular gland had high ^68^Ga-PSMA-11 aggregation, but uptake of ^68^Ga-PSMA-11 in chronic inflammatory atrophy of the submandibular gland was significantly lower [18].

The purpose of this study was to explore the value of ^68^Ga-PSMA-11 PET/CT in evaluating salivary and lacrimal gland function and whether ^68^Ga-PSMA-11 PET/CT has a complementary or alternative role to SGS.

## 2. Materials and Methods

### 2.1. Patient Selection

A total of 15 patients (10 men, 5 women; aged 19–75 years [56.9 ± 15.4 years]) with xerostomia and suspected salivary gland dysfunction were recruited in this study. The study included 4 patients with Sjogren's syndrome, 10 patients with head and neck tumors after radiotherapy and chemotherapy, and 1 patient with surgery for parotid space-occupying lesions. All patients underwent ^68^Ga-PSMA PET/CT first, followed by SGS the next day. The inclusion criteria were as follows: clinical diagnosis of Sjogren's syndrome; radiotherapy and chemotherapy for head and neck tumors; high-dose ^131^I therapy; salivary gland surgery; salivary gland space-occupying lesions; and salivary gland dysplasia. The exclusion criteria were as follows: impairment of liver and kidney function, low white blood cell count, or pregnancy or lactation. The study followed the 1964 Helsinki Declaration and its later amendments or comparable ethical standards.

### 2.2. Image Acquisition

#### 2.2.1. ^68^Ga-PSMA-11 PET/CT

Images were acquired from the skull vertex to the root of neck using a PHILIPS Gemini TF PET/CT 16-tier system approximately 60 minutes after intravenous injection of 1.85MBq/kg ^68^Ga-PSMA-11. Patients did not need specific preparation before PET/CT imaging. A low-dose CT scan (voltage: 120 kV, current: 100 mAs, 5 mm layer, 512 × 512 matrix, and 60 cm FOV) was performed first. Then, PET was performed (9-10 beds, 3 minutes/bed). After reconstruction, image analysis was performed using PHILIPS postprocessing fusion software.

#### 2.2.2. ^99m^TcO_4_^−^ Salivary Gland Scintigraphy

On the day after ^68^Ga-PSMA-11 PET/CT, the patients underwent SGS. The patients were injected with 185 MBq of ^99m^TcO_4_^−^, and dynamic images were acquired using a dual-head gamma camera (GE Healthcare, USA) equipped with a low-energy, high-resolution collimator (128 × 128 matrix, 140 KeV energy peak, 20% window width, 2 times amplification). The anterior image was collected with a probe, and the field of vision covered the entire thyroid and salivary glands. The blood perfusion phase image was collected immediately after intravenous injection of ^99m^TcO_4_^−^ (2 s/frame, 30 frames in total). Then, functional imaging was performed for 30 minutes at 40 s/frame. Vitamin C was given to stimulate salivary secretion 5 minutes before completion of the imaging. Images were analyzed on a Xeleris Workstation (GE Healthcare).

### 2.3. Image Analysis

#### 2.3.1. ^68^Ga-PSMA-11 PET/CT

*Visual analysis*. Three experienced nuclear physicians independently read the PET/CT images. When opinions differed, the majority opinion prevailed. Homogeneous, symmetrical, and strong uptake of ^68^Ga-PSMA-11 in the bilateral parotid and submandibular glands was defined as normal. Abnormal uptake of ^68^Ga-PSMA-11 in salivary glands included one of the following conditions. (a) The level of ^68^Ga-PSMA-11 uptake in salivary glands was similar to that in background tissues (e.g., subcutaneous soft tissue of the head and neck); and (b) ^68^Ga-PSMA-11 uptake into the glands presents a visible decrease compared to the contralateral side. *Quantitative analysis*. To quantify ^68^Ga-PSMA-11 uptake, the volumes of interest (VOIs) of the bilateral lacrimal glands, parotid glands, submandibular glands, and thyroid glands were mapped on serial images. The maximum standardized uptake value (SUVmax) in the VOI of each gland was obtained by the software.

#### 2.3.2. ^99m^TcO_4_^−^ Salivary Gland Scintigraphy

Visual evaluation: the images were independently and visually assessed by three experienced nuclear physicians. When the opinions differed, the majority opinion prevailed. The physicians were unaware of the results of other clinical studies. Classification of salivary gland function was based on the criteria used in the study by Kim et al. [19]. Salivary gland uptake is considered normal when it is similar to thyroid uptake. Normal excretion is defined as salivary gland activity similar to background activity after stimulation. Salivary gland function was graded on a scale from 1 to 3: 1, normal; 2, mild-to-moderate; and 3, severe. Grade 1 indicated that the uptake and excretion of salivary glands were normal; grade 2 indicated low uptake and/or delayed excretion; and grade 3 was defined as severe dysfunction with a complete absence of radioactivity in the salivary glands. Semiquantitative analysis: semiquantitative analysis was performed using the region of interest (ROI) technique. The ROIs of the bilateral parotid gland and submandibular gland were depicted, and the bilateral temporal region was used as a background reference. The computer automatically plotted the time-activity curves. From the time-activity curves, the maximum value before vitamin C administration, the minimum value after vitamin C administration, and the background uptake value were obtained. The uptake ratio (UR) and excretion fraction (EF) of each salivary gland were calculated according to the following equation: UR = (maximum-background uptake)/background uptake; EF = (maximum-minimum)/(maximum-background uptake) × 100% [20]. Decreased uptake function for the respective glands was defined as follows: UR (parotid gland) <2.28 and UR (submandibular gland) <1.60 [19]. Abnormality was indicated if the criteria were met in either visual analysis or semiquantitative analysis.

## 3. Results

The general information and PET and SGS imaging results for all 15 patients are shown in Table 1. This study included 4 patients with Sjogren's syndrome (patients 1–4), 10 patients with head and neck tumors after radiotherapy and chemotherapy (patients 5–14), and 1 patient with surgery for parotid space-occupying lesions (patient 15).

### 3.1. Image Findings by ^68^Ga-PSMA-11 PET/CT and ^99m^TcO_4_^−^ Salivary Gland Scintigraphy

The results of ^68^Ga-PSMA-11 PET/CT and SGS were consistent in 12/15 patients (80%; patients 1–12). Four patients with Sjogren's syndrome and 4 patients with head and neck cancer after radiotherapy and chemotherapy showed positive results, whereas four patients with head and neck cancer after radiotherapy and chemotherapy showed negative results.

The results of the two examinations were not consistent with those in the other 3 patients (patients 13–15). One patient who underwent surgery for parotid space-occupying lesions was positive only on ^68^Ga-PSMA-11 PET/CT, which corrected the misdiagnosis by SGS. Two patients with head and neck cancer after radiotherapy and chemotherapy were positive only on SGS.

In 2 (13.3%; patients 5 and 15) of 15 patients, ^68^Ga-PSMA-11 PET/CT provided more accurate information about the salivary glands than did SGS.

Additionally, in 3 (20%; patients 1–3) of 15 patients, ^68^Ga-PSMA-11 PET/CT provided information about the lacrimal gland; decreased uptake of ^68^Ga-PSMA-11 was observed.

**Figure 1:**
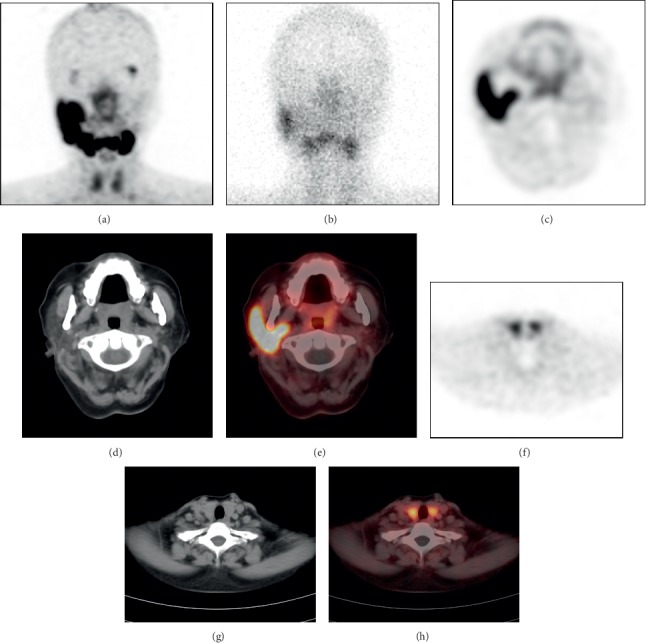
A 55-year-old woman (patient 1) with dry mouth and a dry right eye for half a year was initially diagnosed with sjogren's syndrome. ^68^Ga-PSMA-11 PET/CT MIP (a) and tomographic images (c–e) showed that the left parotid gland (SUVmax, 1.7) was smaller in volume and lower in density without ^68^Ga-PSMA-11 uptake than the right parotid gland (SUVmax, 9.2). SGS (b) confirmed that the uptake function of the left parotid gland was severely decreased (UR_left parotid_, 2.5; UR_right__parotid_, 10.0). In addition, it was found that the ^68^Ga-PSMA-11 uptake of the right lacrimal gland was lower than that of the left side (SUVmax_right__lacrimal gland_, 2.0; SUVmax_left__lacrimal gland_, 4.2). Combined with ^68^Ga-PSMA-11 PET/CT and the patient's medical history, the uptake function of the right eye was considered to be reduced. It was also found that the thyroid (f–h) density decreased with ^68^Ga-PSMA-11 diffuse uptake but without ^99m^TcO_4_^−^ uptake. The patient with a history of taking supplements had a 7-year history of Hashimoto's thyroiditis.

Among all 15 patients, only one had an incidental finding of diffuse uptake of ^68^Ga-PSMA-11 in the thyroid (patient 1: Figure 1, SUVmax 4.6). No significant uptake of ^68^Ga-PSMA-11 was observed in the thyroids of the other patients. This patient was diagnosed with Hashimoto's thyroiditis.

## 4. Discussion

Klein et al. assessed the physiological distribution of ^68^Ga-PSMA-11 in the salivary glands and seromucous glands of the head and neck and observed increased tracer uptake in all gland locations. The mean SUVmax ± standard deviation varied as follows: parotid, 12.3 ± 3.9 (range 5.2–22.9); submandibular gland, 11.7 ± 3.5 (range 6.0–22.2); sublingual gland, 4.5 ± 1.9 (range 1.2–8.5); and lacrimal gland, 6.2 ± 2.2 (range 2.5–13.6) [16]. Our study also confirmed the high uptake of ^68^Ga-PSMA-11 in the salivary and lacrimal glands, which is consistent with previous studies [14–17].

**Figure 2:**
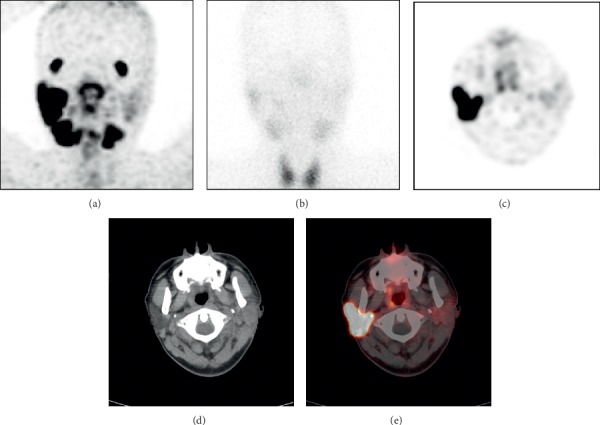
A 50-year-old male (patient 15) underwent left parotid gland resection for a left parotid mass one month previously. Postoperative pathology revealed left parotid acinar cell carcinoma. ^68^Ga-PSMA-11 PET/CT maximum density projection (MIP) (a) and corresponding tomography (c–e) show the absence of the left parotid gland and structural disorder of the soft tissue with slight uptake of ^68^Ga-PSMA-11 (SUVmax, 2.7; right parotid gland SUVmax, 20.8). SGS (b) shows that ^99m^TcO_4_^−^ uptake in the left parotid gland region (UR 4.3) was slightly lower than that in the right parotid gland (UR 5.6). In addition, the thyroid uptake of ^99m^TcO_4_^−^ was significantly increased; this patient had a history of hyperthyroidism and took propylthiouracil for half a year.

In our study, the results of ^68^Ga-PSMA-11 PET/CT and SGS were consistent in 12 patients and inconsistent in the other 3 patients. Moreover, ^68^Ga-PSMA-11 PET/CT corrected the misdiagnosis of patient 15 (Figure 2) by SGS and provided more information for 5 patients (33%) than SGS. ^68^Ga-PSMA-11 PET/CT showed promising potential in evaluating the function of the lacrimal gland and salivary gland.

**Figure 3:**
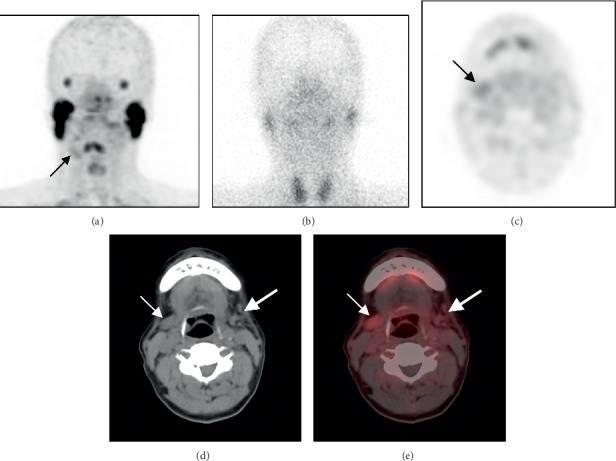
A 64-year-old male patient (patient 5) with nasopharyngeal carcinoma received radiotherapy and chemotherapy. The ^68^Ga-PSMA-11 PET/CT MIP (a) and tomographic images (c–e) clearly show a slight uptake of ^68^Ga-PSMA-11 in the right submandibular gland (small arrow; SUVmax, 2.6) but no significant uptake of imaging agents in the left submandibular gland (large arrow; SUVmax, 1.9). The slice images (d–e) show that the volume of the left submandibular gland was significantly reduced. SGS (b) showed no significant ^99m^TcO_4_^−^ uptake in the bilateral submandibular glands, confirming a severe decrease in its uptake function.

SGS is a planar SPECT imaging technique, while ^68^Ga-PSMA-11 PET/CT is a positron emission computed tomography fusion imaging technique. ^68^Ga-PSMA-11 PET/CT can provide functional metabolic and anatomical images with higher resolution than SGS. ^68^Ga-PSMA-11 PET/CT can accurately assess the morphology of the gland and the degree of atrophy to evaluate gland function more accurately. For example, in patient 1 (Figure 1) and patient 5 (Figure 3), ^68^Ga-PSMA-11 PET/CT clearly shows the degree of atrophy and the density changes in the parotid and submandibular glands. In addition to the inherent advantages of PET/CT, ^68^Ga-PSMA-11 PET/CT has the following unique advantages in the evaluation of lacrimal and salivary gland function.

- *^68^Ga-PSMA-11 PET/CT is significantly less affected by surgery than SGS*. For example, in patient 15 (Figure 2), the left parotid gland region showed significant uptake of ^99m^TcO_4_^−^ and slight uptake of ^68^Ga-PSMA-11. The ^68^Ga-PSMA-11 PET/CT axial images clearly showed the absence of the left parotid gland and the structural disorder of the soft tissue. The ^99m^TcO_4_^−^ uptake in the left parotid gland region was considered to be caused by the operation according to the patient's history of total parotid gland resection one month previously. Without an accurate medical history, it may be a mistake to presume that the uptake function of the left parotid gland is normal. However, ^68^Ga-PSMA-11 PET/CT can accurately show the postoperative changes in the left parotid gland and is significantly less affected by surgery.
- *^68^Ga-PSMA-11 PET/CT has higher sensitivity and image contrast for the uptake of imaging agents in glands than SGS*. For example, on the ^68^Ga-PSMA-11 PET/CT images, patient 5 showed slight uptake of ^68^Ga-PSMA-11 in the right submandibular gland (Figure 3(a, c–e) small arrows, SUVmax, 2.6) and differences in the bilateral submandibular glands (SUVmax, right: left = 2.6 : 1.9). It could not be observed on the SGS image (Figure 3(b)). The bilateral parotid glands of patient 1 (Figure 1) showed better contrast on ^68^Ga-PSMA-11 PET/CT (SUVmax_right_: SUVmax_left_ = 9.2 : 1.7 = 5.4 : 1) than that on SGS (UR_right_: UR_left_ = 10.0 : 2.5 = 4 : 1). ^68^Ga-PSMA-11 PET/CT can clearly show the uptake of the imaging agent in the salivary glands, even in cases of low levels of imaging agents that are not normally visible on SGS. ^68^Ga-PSMA-11 PET/CT can more clearly show and provide better image contrast for visible and invisible differences in gland pairs on SGS.
- *The visual analysis of SGS imaging is affected by the extent of thyroid imaging agent uptake, but^68^Ga-PSMA-11 PET/CT image analysis is not affected. In addition,^68^Ga-PSMA-11 PET/CT can also unexpectedly identify thyroid lesions*. Visual analysis of SGS images is usually based on the level of imaging agent uptake in the thyroid gland. The thyroid uptake of ^99m^TcO_4_^−^ increased in patient 15 (Figure 2). At this time, image analysis based on the thyroid uptake level underestimates the uptake function of salivary glands. No significant ^99m^TcO_4_^−^ uptake was observed in the thyroid of patient 1 (Figure 1(b)), and visual analysis of salivary gland function relied on the experience of the observer. ^68^Ga-PSMA-11 PET/CT images can be directly visualized and quantitatively analyzed without the thyroid as a reference. In patient 1 (Figure 1(f)–1(h)), diffuse uptake of ^68^Ga-PSMA-11 in the thyroid was unexpectedly found but without ^99m^TcO_4_^−^ uptake on the corresponding SGS imaging. The patient was diagnosed with Hashimoto's thyroiditis. A study by Kirchner, J et al. found that diffuse uptake in the thyroid (SUVmax ± SD: 4.5 ± 1.2) of ^68^Ga-PSMA-11 was found in 22% (12/55) of patients. However, there were no indications about thyroid malignancies or other serious diseases in their data [14]. Some reports described the uptake of ^68^Ga-PSMA in follicular adenoma, differentiated thyroid cancer, and medullary thyroid cancer [21–23]. A study by Silver et al. showed no expression of PSMA in the thyroid gland [24]. A previous study has shown that neovascular PSMA expression is common in thyroid cancer but may also rarely be found in benign thyroid diseases, such as follicular adenoma [25].
- *^68^Ga-PSMA-11 PET/CT can provide accurate localization and quantitative analysis for lacrimal glands. This is not possible on SGS*. Three patients showed decreased uptake of ^68^Ga-PSMA-11 in their lacrimal glands. For example, in patient 1 (Figure 1), the uptake of lacrimal gland ^68^Ga-PSMA-11 on the right side was lower than that on the left side (SUVmax, right:left = 2.0 : 4.2). Combined with the patient's symptom of right eye dryness and the diagnosis of Sjogren's syndrome, the uptake function of the right lacrimal gland was considered to be reduced. SGS can only evaluate the morphology and function of the parotid and submandibular glands, but it is useful for imaging and observing small glands, such as the lacrimal gland and sublingual gland. Our study shows that ^68^Ga-PSMA-11 PET/CT can be used to visualize the lacrimal gland and evaluate its function.

**Figure 4:**
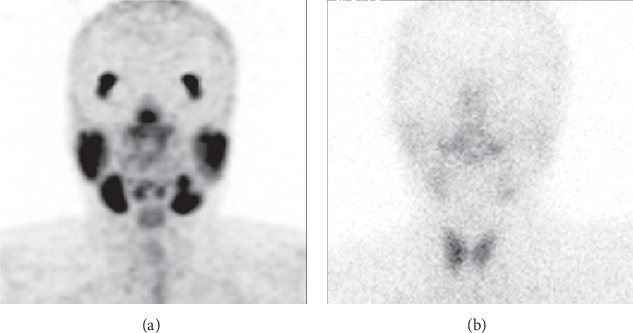
A 43-year-old female (patient 14) with nasopharyngeal carcinoma after radiotherapy and chemotherapy. ^68^Ga-PSMA-11 PET/CT MIP (a) shows that the imaging agents were evenly and symmetrically distributed in bilateral lacrimal glands and major salivary glands (right parotid gland, left parotid gland, right submandibular gland, and left submandibular gland; SUVmax values of 8.1, 8.0, 10.2, and, 8.2, respectively). No obvious abnormalities were observed. However, ^99m^TcO_4_^−^ SGS (b) shows a mild-to-moderate decrease in the uptake function of the left parotid (right parotid gland, left parotid gland, right submandibular gland, and left submandibular gland; UR values of 3.1, 2.2, 3.4, and 2.8, respectively).

However, ^68^Ga-PSMA-11 PET/CT imaging also has the following limitations in assessing salivary gland function. *(a)^68^Ga-PSMA-11 PET/CT cannot evaluate the excretion function of salivary glands*. There is no effective method to reduce PSMA ligand uptake in salivary glands [26]. Further research is warranted. *(b) Visual evaluation of^68^Ga-PSMA-11 PET/CT may have limitations in patients with mild to moderate reduction of salivary gland uptake on SGS*. In patient 14 (Figure 4), SGS showed a mild-to-moderate decrease in bilateral parotid and submandibular gland uptake. However, there was no significant abnormality on ^68^Ga-PSMA PET/CT imaging. This may be a defect in the visual assessment of the ^68^Ga-PSMA-11 PET/CT, which may be compensated by more accurate quantitative analysis criteria.

Salivary gland analysis in ^68^Ga-PSMA-11 PET/CT still has some problems that need to be solved. *(a) The uptake mechanism of^68^Ga-PSMA-11 in salivary glands is unclear*. Tracer uptake in prostate cancer is based on the expression of PSMA receptors in cells. However, the previous literature shows that the mechanism of the intense accumulation of PSMA radioligands in salivary glands is unclear [18]. Normal salivary glands and lacrimal glands have high PSMA ligand uptake. It was previously reported that PSMA is physiologically expressed in normal salivary glands but at much lower levels than those in prostate cancer tissue [18, 27]. There are also studies showing that there is no PSMA expression in salivary glands [24, 28]. The intense uptake of PSMA ligands in salivary glands is not correlated with the expression level of PSMA. Some studies have shown that the uptake of PSMA-targeted radioligands in salivary gland tissues may be caused by both nonspecific and PSMA-specific uptake. However, the exact proportion of each form of uptake is still uncertain, and the mechanism of the nonspecific uptake is still unclear [29–32]. Recently, it has been reported that the accumulation of PSMA-targeted radioligands in salivary gland tissue is mainly caused by nonspecific uptake [18, 33]. Further research will be necessary to investigate the exact mechanism of radioligand accumulation in salivary glands. *(b) At present, there is no uniform classification standard for^68^Ga-PSMA-11 uptake in lacrimal glands and major salivary glands*. The clinical experience of ^68^Ga-PSMA-11 PET/CT image interpretation is still limited. The uptake patterns of ^68^Ga-PSMA-11 in salivary and lacrimal glands may need to be analyzed in future large sample studies and to develop accurate and reliable classification criteria.

## 5. Conclusions

^68^Ga-PSMA-11 PET/CT imaging shows promising results in terms of lacrimal gland and major salivary gland uptake function. In contrast to SGS, ^68^Ga-PSMA-11 PET/CT can visualize small glands, such as the lacrimal gland and sublingual gland, and is less affected by surgery. ^68^Ga-PSMA-11 PET/CT may be used as a supplementary examination tool for SGS in the evaluation of salivary gland function. This study is a preliminary exploratory study, and the results of this study still need to be verified in subsequent large-scale studies.

## Figures and Tables

**Table 1:** Patient data and analysis of the uptake characteristics of ^99m^TcO_4_^−^ and ^68^Ga-PSMA-11 in all 15 patients.

Patient no.	Sex	Age (Y)	History	Abnormal uptake of ^99m^TcO_4_^−^ in PG or SM	Abnormal uptake of ^68^Ga-PSMA-11 in PG or SM	Additional findings from ^68^Ga-PSMA-11 PET/CT
1	F	55	Sjogren's syndrome	Lt. PG	Lt. PG	Rt. LG, T
2	F	67	Sjogren's syndrome	Rt. SM, Lt. SM	Rt. SM, Lt. SM	Rt. LG, Lt. LG
3	M	71	Sjogren's syndrome	Rt. PG, Lt. PG	Rt. PG, Lt. PG	Rt. LG, Lt. LG
4	M	70	Sjogren's syndrome	Rt. SM	Rt. SM	—
5	M	64	NPC after radiotherapy and chemotherapy	Rt. SM, Lt. SM	Rt. SM, Lt. SM	Rt. SM
6	M	64	Vocal cord cancer after radiotherapy and chemotherapy	Lt. PG, Lt. SM	Lt. PG, Lt. SM	—
7	F	41	NPC after radiotherapy and chemotherapy	Lt. PG	Lt. PG	—
8	F	66	NPC after radiotherapy and chemotherapy	Rt. SM	Rt. SM	—
9	M	75	Papillary carcinoma of the jaw after radiotherapy and chemotherapy	N	N	—
10	M	72	Vocal cord cancer after radiotherapy and chemotherapy	N	N	—
11	M	46	NPC after radiotherapy and chemotherapy	N	N	—
12	M	19	EMP of nasal cavity after radiotherapy and chemotherapy	N	N	—
13	M	50	NPC after radiotherapy and chemotherapy	Rt. SM	N	—
14	F	43	NPC after radiotherapy and chemotherapy	Lt. PG	N	—
15	M	50	Postoperative treatment of left parotid acinar cell carcinoma	N	Lt. PG	Lt. PG

NPC = nasopharyngeal carcinoma; EMP = extramedullary plasmacytoma; N = negative; LG = lacrimal gland; PG = parotid gland; SM = submandibular gland; T = Thyroid. “—” means no relevant data.

## Data Availability

The data used to support the findings of this study are included within the article.
